# Influence of the home smoking environment and stress on smoking behaviors during the COVID-19 pandemic among patients of federally qualified health centers in rural Georgia

**DOI:** 10.18332/tpc/195832

**Published:** 2024-12-10

**Authors:** Michelle C. Kegler, Angela Zhang, Regine Haardörfer, Ja'Shondra Pouncy, Crystal Owens, Carla J. Berg

**Affiliations:** 1 Department of Behavioral, Social and Health Education Sciences, Emory Prevention Research Center, Rollins School of Public Health, Emory University, Atlanta, United States; 2 Community Health Care Systems, Tennille, United States; 3 Department of Prevention and Community Health, Milken Institute School of Public Health, George Washington University, Washington, United States; 4 GW Cancer Center, George Washington University, Washington D.C., United States

**Keywords:** smoking behavior, tobacco use, COVID-19, rural, cessation attempts

## Abstract

**INTRODUCTION:**

The COVID-19 pandemic caused major stress, as well as changes to home and work environments, with the potential to alter smoking-related behaviors. This study examined determinants of smoking-related behaviors among patients of federally qualified health centers (FQHCs) in Georgia.

**METHODS:**

We analyzed survey data from 353 patients (mean age=50 years, 62.9% women, 54.4% Black/African American, 27.8% <high school education, 56.5% ≤$25000 annual income) enrolled in a smoking cessation trial, from 3 FQHCs in rural Georgia (November 2020 to December 2022). Multivariable multinomial regression examined home smoking environments (i.e. household members who smoke, household smoking rules) and changes in stress in relation to: 1) smoking increases/decreases in general and in the home, and 2) quit attempts since pre-pandemic.

**RESULTS:**

Most study participants (85.6%) smoked daily, and 41.6% had smoke-free homes. Compared to pre-pandemic, 36.3% reported increased stress, 28.8% increased smoking, 18.8% increased in-home smoking, and 55.4% quit attempts. Regression models showed more household members who smoke (AOR=1.56; 95% CI: 1.02–2.39) and greater stress (AOR=5.52; 95% CI: 2.74–11.12) were associated with increased smoking (vs no change) since the pandemic began. Non-daily (vs daily) smoking (OR=4.79; 95% CI: 1.71–13.46) was associated with decreased smoking (vs no change). Allowing smoking in the home and greater stress were associated with both increased and decreased in-home smoking (vs no change). We found no associations with quit attempts.

**CONCLUSIONS:**

Home environments, specifically home smoking restrictions, as well as stress, may be important intervention targets during societal stressors.

## INTRODUCTION

The COVID-19 pandemic was stressful for many, with fear of sickness and death, disrupted employment, financial hardship, altered work schedules, changed work environments with remote working, children at home during the day with school closures, less socializing with extended family and friends, and boredom^1-4^. Studies have shown its varied influence on a range of health behaviors, including smoking, with some showing the majority of those who smoke did not change their consumption levels^5-8^, and others showing reduced^9-11^ or increased smoking^11-13^. A review of 77 studies worldwide, reported that increased smoking during COVID-19 lockdowns in 2020 was most common, although a substantial proportion showed no change and decreases^11^. In contrast, a review of 11 longitudinal studies reported that most documented reduced cigarette consumption from before to during the pandemic^14^.

Studies vary substantively in timing, methods, measures, and populations, making it difficult to conclude who changed their behavior and why^11,14^ . Both qualitative and quantitative studies, however, have demonstrated the influence of stress and psychological distress on increased smoking for some^4,7,13,14^. Studies have also linked financial problems to increased smoking^13,15^. Qualitative studies suggest that changes in environments (e.g. less time at work where smoking is restricted, more time at home, and boredom) may have also contributed to increased smoking^4,16,17^. Reasons for decreased cigarette consumption center on fear of COVID-19^14,18^, sometimes exacerbated by concerns of increased vulnerability due to weakened lungs from smoking^4,5,19^. Financial strain and less socializing with friends who smoke also led some individuals to smoke less^4,17,20^. A few studies examined whether COVID-19 contributed to increased quit attempts or successful cessation. A study in England reported that a small proportion of quit attempts were due to COVID-19^21^, while others showed increased interest or attempts due to COVID-19^4,13,16,18^. Interestingly, an analysis of past-year quit attempts comparing 2011–2019 to 2020 using national data from the US, showed decreased cessation attempts^22^.

Several US subpopulations faced COVID-19-related disparities, including those with lower socioeconomic status, rural communities, and racial/ethnic minorities^20,23^. Because these populations also experience a disproportionate burden of tobacco-related morbidity and mortality^24-28^, it is crucial to understand how the COVID-19 pandemic and related socio-contextual factors impacted smoking-related behaviors in these key populations.

Federally qualified health centers (FQHCs) serve as the primary care safety net for the US, providing care for many people who live in poverty and experience significant inequities^29^. This study examined the influence of COVID-19 and related stress, as well as household smoking context (household smoking rules, number of household members who smoke), on cigarette consumption in general and in the home, along with quit attempts among FQHC patients who smoke in rural Georgia. We hypothesized that persons who experienced increased stress, lived with a greater number of household members who smoke and allowed smoking in the home, were more likely to increase smoking levels during the pandemic. Findings from this study may aid in efforts to promote positive changes in smoking-related behaviors during societal stressors.

## METHODS

This analysis uses baseline data from an ongoing randomized controlled trial testing the efficacy of integrating an evidence-based smoke-free home intervention^30^ into the 5As approach for tobacco cessation in FQHCs. Data were collected from mid November 2020 to mid December 2022. Healthcare providers from three FQHCs serving rural Georgia referred their interested patients who smoked. Eligibility criteria included being aged ≥18 years, having smoked at least one cigarette in the past 30 days, and speaking English. The Emory University Institutional Review Board approved the research protocol and materials. Verbal consent was obtained prior to completing a telephone-administered baseline survey.

### Measures

*Independent variables: household smoking context, COVID-related stress level, and smoking level*

Participants were asked how many people lived in the home, how many were children aged <5 years, and how many household members smoked cigarettes. To assess household smoking rules, participants were asked: ‘Which statement best describes the rules about smoking inside your home? (This does not include decks, garages, or porches)’. Response options were smoking is not allowed anywhere inside your home; there are no rules about smoking inside your home; smoking is allowed in some places or at some times; or smoking is allowed anywhere inside your home^31,32^. To assess pandemic-related stress levels, participants were asked: ‘Since COVID-19 began, is your stress level: better, about the same, or worse?’. Participants were also asked if they currently smoked every day or on some days.

*Dependent variables: change in smoking behaviors since pre-COVID-19*

Changes in smoking levels were assessed by asking: ‘Since the COVID-19 pandemic began, did your [cigarette smoking/number of cigarettes smoked inside your home] decrease, stay the same, or increase?’. Participants were also asked if they had tried to quit smoking since the COVID-19 pandemic began (yes/no).

*Descriptive variables: COVID-related stressors, smoking-related factors, and housing type*

Specific COVID-19-related stressors were assessed by asking if they had been affected by six different stressors since the pandemic began (e.g. you have been affected by additional family members moving in; self or household member laid off from work). Participants were also asked about their housing type (i.e. single-family home, apartment building, duplex, townhome), cigarette purchasing (how are you managing to purchase cigarettes: same as always, or it has changed), and whether they had tried to cut down on smoking cigarettes since the pandemic began (yes/no).

*Covariates: demographics*

Sex, age, marital status, race/ethnicity, education level, annual pre-tax household income, employment status, and number of people in the household, were assessed.

### Data analysis

Descriptive analyses were conducted to characterize independent variables, dependent variables, and covariates. Bivariate analyses examining independent variables of interest (household smoking context, worsened stress since the COVID-19 pandemic began) and covariates in relation to each dependent variable (i.e. increase or decrease [vs no change] in cigarette smoking in general and in the home since pre-COVID-19, any quit attempt since pre-COVID-19) used unadjusted multinomial logistic regression for changes in cigarette smoking overall and in the home, respectively (i.e. increases or decreases, vs no change) and binary logistic regression for quit attempts (yes vs no). We then estimated multivariable multinomial regression models to investigate associations between independent variables and changes in overall cigarette smoking, smoking in the home, and quit attempts since pre-COVID-19. Potential covariates significant at p<0.05 in any of the bivariate models were included in all multivariable multinominal models. All analyses were performed using SAS version 9.4.

## RESULTS

### Descriptive analyses

As shown in Table 1, the mean age of participants was 50 years (SD=12.49). Over half were women (62.9%), identified as Black (54.4%), and had a high school education (72.2%). The mean household size was three persons (SD=1.54), with an average of two persons who smoked (SD=0.80); 12.5% of households included a child aged <5 years. The majority lived in a single-family home (63.9%). Less than half (41.6%) reported having a smoke-free home.

**Table 1:** Descriptive analyses of dependent variables, independent variables, and sociodemographic covariates among FQHC patients in rural Georgia (N=353)

	*n*	*%*
**Dependent variables**		
**Cigarette smoking in general^a^**		
Increased	101	28.8
Decreased	64	18.2
Stayed the same	186	53.0
**Number of cigarettes smoked inside home^a^**		
Increased	66	18.8
Decreased	45	12.8
Stayed the same	240	68.4
**Tried to quit smoking cigarettes**	195	55.4
**Independent variables**		
**Number of smokers in home**, mean (SD)	2 (0.80)	
**Home smoking rules**		
Not allowed anywhere	147	41.6
Allowed some places or some times	89	25.2
Allowed anywhere/no rules	117	33.1
**Stress level**		
Worse	128	36.3
Not worse	225	63.7
**Smoking level**		
Daily smoking	302	85.6
Non-daily smoking	51	14.5
**Sociodemographic covariates**		
**Sex**		
Male	128	36.3
Female	222	62.9
Other	3	0.8
**Age** (years), mean (SD)	50 (12.49)	
**Marital status**		
Married or cohabiting	158	44.8
Single	195	55.2
**Race/ethnicity**		
White	139	39.4
Black/African American	192	54.4
Multi-racial/Mixed	8	2.3
Other	14	4.0
**Education level**		
Some high school or lower	98	27.8
High school graduate or GED	141	39.9
Vo-tech or some college	73	20.7
College graduate or higher	41	11.6
**Annual household income** (US$)		
≤25000	199	56.5
25001–50000	66	18.8
50001–75000	22	6.3
>75000	25	7.1
Don’t know/refused	40	11.4
**Number of people in home**, mean (SD)	3 (1.54)	

a Denominators vary slightly with exclusion of ‘don’t know’ and missing responses.

The majority (55.1%) reported that their stress levels remained the same, although 36.3% reported increased stress since the pandemic began and 7.7% reported less stress. Specific stressors included: greater difficulty paying utilities (44.5%), rent/house payments (35.4%), and food (42.5%); started using a food pantry (33.7%); having children at home with school/daycare closing (26.7%); someone in the household being laid off from work (21.0%); and having additional family members move in (13.9%).

Over half (53.0%) reported their cigarette smoking had stayed the same since the pandemic began, although 28.8% reported increases and 18.2% decreases. Similarly, the majority (68.4%) reported no change in the number of cigarettes smoked inside the home, which most commonly stayed the same or was viewed as not applicable (e.g. due to a smoke-free home rule). In comparison, 18.8% reported increases and 12.8% decreases. The majority had tried to quit smoking (55.4%) or cut down (77.5%) as a result of the pandemic, but relatively few (11.2%) had changed their cigarette purchasing habits.

### Associations with smoking levels in general and in the home since the COVID-19 pandemic began

*Smoking levels in general*

In bivariate analyses, the number of household members who smoke and worsened stress levels were associated with increased smoking versus no change; non-daily smoking, identifying as Black/African American, and an annual household income ≤$25000 were associated with less smoking versus no smoking. In multivariable, multinomial models, a higher number of household members who smoke (AOR=1.56; 95% CI: 1.02–2.39) and worsened stress levels (AOR=5.52; 95% CI: 2.74–11.12) were associated with increased smoking since the pandemic began (Table 2).

**Table 2:** Multinomial regression showing associations between household smoking context, participant characteristics and changes in cigarette smoking versus no change since pre-COVID-19 among FQHC patients in rural Georgia

*Variables*	*Smoking increased (N=101) vs No change (N=186)*		*Smoking decreased (N=64) vs No change (N=186)*		*Smoking increased (N=71) vs No change (N=114)*		*Smoking decreased (N=39) vs No change (N=114)*	
	*OR*	*(95% CI)*	*OR*	*(95% CI)*	*AOR*	*(95% CI)*	*AOR*	*(95 % CI)*
**Independent variables**								
**Number of smokers in home**	**1.61**	**(1.14–2.28)**	1.19	(0.77–1.83)	**1.56**	**(1.02–2.39)**	0.94	(0.49–1.81)
**Home smoking rules**								
Not allowed anywhere ®	1		1		1	1		
Allowed some places or some times	1.14	(0.61–2.12)	0.96	(0.47–1.96)	1.03	(0.45–2.35)	0.51	(0.18–1.47)
Allowed anywhere/no rules	1.50	(0.85–2.63)	1.02	(0.52–1.98)	1.47	(0.64–3.39)	0.70	(0.23–2.14)
**Stress level**								
Worse	**3.69**	**(2.22–6.15)**	0.95	(0.50–1.81)	**5.52**	**(2.74–11.12)**	1.93	(0.78–4.82)
Not worse ®	1		1		1		1	
**Smoking level**								
Daily smoking ®	1		1		1		1	
Non-daily smoking	0.73	(0.32–1.65)	**3.15**	**(1.57–6.32)**	0.997	(0.35–2.84)	**4.79**	**(1.71–13.46)**
**Sociodemographic covariates**								
**Sex**								
Male ®	1		1					
Female	1.45	(0.87–2.43)	1.13	(0.63–2.05)				
**Age** (years)	0.99	(0.97–1.01)	1.01	(0.99–1.04)				
**Marital status**								
Married or cohabiting ®	1		1		1	1		
Single	0.80	(0.50–1.31)	1.15	(0.65–2.05)	0.68	(0.33–1.41)	**0.36**	**(0.14–0.92)**
**Race/ethnicity**								
White ®	1		1		1	1		
Black/African American	1.07	(0.65–1.76)	**1.99**	**(1.06–3.72)**	1.67	(0.81–3.45)	2.56	(0.99–6.63)
**Education level**								
Some high school or lower	0.99	(0.44–2.23)	3.19	(0.99–10.28)	1.01	(0.32–3.17)	2.47	(0.51–12.01)
High school graduate or GED	0.82	(0.38–1.77)	1.70	(0.53–5.40)	1.10	(0.38–3.13)	1.14	(0.24–5.36)
Vo-tech or some college	0.86	(0.37– 2.02)	1.73	(0.50–5.98)	1.04	(0.34–3.11)	0.70	(0.12–4.19)
College graduate or higher ®	1		1		1		1	
**Annual household income (US$)**								
≤25000	1.72	(0.82–3.59)	**8.34**	**(1.92–36.26)**	2.13	(0.81–5.59)	**9.47**	**(1.79–49.99)**
25001–50000	1.31	(0.56–3.06)	1.57	(0.27–9.11)	0.81	(0.29–2.30)	1.53	(0.24–9.82)
>50000 ®	1		1		1	1		

AOR: adjusted odds ratio. ® Reference categories.

An annual household income ≤$25000 was associated with greater odds of decreased smoking (AOR=9.47; 95% CI: 1.79–49.99), as was non-daily smoking (AOR=4.79; 95% CI: 1.71–13.46). Being single (AOR=0.36; 95% CI: 0.14–0.92) was associated with lower odds of decreased smoking since the pandemic began.

*Smoking levels in the home*

In bivariate analyses, partial or no smoking restrictions and worsened stress levels were associated with increased smoking in the home relative to no change. Lack of restrictions, non-daily smoking, being single, and identifying as Black/African American were associated with decreased smoking inside the home since the pandemic began relative to no change.

Allowing smoking in the home, either under a partial ban (AOR=10.58; 95% CI: 3.21–34.85) or no restrictions (AOR=11.08; 95% CI: 3.19–38.53), was associated with increased smoking inside the home since the pandemic, as were identifying as Black/African American (AOR=3.01; 95% CI: 1.13–8.02) and a worsened stress level (AOR=8.29; 95% CI: 3.24–21.23) in multivariable models. Similarly, a partial ban (AOR=4.04; 95% CI: 1.28–12.76) and no restrictions (AOR=4.06; 95% CI: 1.18–13.99) were associated with decreased smoking inside the home, as were identifying as being Black/African American (AOR=3.85; 95% CI: 1.23–11.99) and worsened stress (AOR=2.81; 95% CI: 1.05–7.57) (Table 3).

**Table 3:** Multinomial regression showing associations between household smoking context, participant characteristics and changes in cigarette smoking inside the home versus no change since pre-COVID-19 among FQHC patients in rural Georgia

*Variables*	*Number of cigarettes smoked inside home increased (N=66) vs No change (N=240)*		*Number of cigarettes smoked inside home decreased (N=45) vs No change (N=240)*		*Number of cigarettes smoked inside home increased (N=39) vs No change (N=156)*		*Number of cigarettes smoked inside home decreased (N=28) vs No change (N=156)*	
	*OR*	*(95% CI)*	*OR*	*(95% CI)*	*AOR*	*(95% CI)*	*AOR*	*(95% CI)*
**Independent variables**								
**Number of smokers in home**	**1.26**	**(0.87–1.83)**	**1.07**	**(0.67–1.69)**	**1.18**	**(0.67–2.07)**	**1.04**	**(0.54–2.02)**
**Home smoking rules**								
Not allowed anywhere ®	1		1		1		1	
Allowed some places or some times	**9.24**	**(3.90–21.89)**	**4.84**	**(2.06–11.33)**	**10.58**	**(3.21–34.85)**	**4.04**	**(1.28–12.76)**
Allowed anywhere/no rules	**7.64**	**(3.34–17.51)**	**3.44**	**(1.50–7.87)**	**11.08**	**(3.19–38.53)**	**4.06**	**(1.18–13.99)**
**Stress level**								
Worse	**4.04**	**(2.28–7.15)**	1.50	(0.77–2.91)	**8.29**	**(3.24– 21.23)**	**2.81**	**(1.05–7.57)**
Not worse ®	1		1	1	1			
**Smoking level**								
Daily smoking ®	1		1		1		1	
Non-daily smoking	0.65	(0.26–1.63)	**2.36**	**(1.11–5.05)**	0.67	(0.18–2.57)	2.45	(0.81–7.42)
**Sociodemographic covariates**								
**Sex**								
Male ®	1		1					
Female	1.07	(0.60–1.90)	1.06	(0.55–2.07)	-	-	-	-
**Age** (years)	1.01	(0.99–1.04)	1.01	(0.998–1.04)	-	-	-	-
**Marital status**								
Married or cohabiting ®	1		1		1		1	
Single	1.01	(0.58–1.73)	**2.19**	**(1.10–4.38)**	0.77	(0.32–1.89)	1.12	(0.42–2.98)
**Race/ethnicity**								
White ®	1		1		1		1	
Black/African American	1.41	(0.79–2.50)	**3.37**	**(1.54–7.34)**	**3.01**	**(1.13–8.02)**	**3.85**	**(1.23– 11.99)**
**Education level**								
Some high school or lower	2.50	(0.87–7.23)	1.74	(0.58–5.20)	3.41	(0.58–20.06)	0.96	(0.23–4.06)
High school graduate or GED	1.40	(0.49–3.98)	0.97	(0.33–2.87)	4.63	(0.83–25.96)	0.73	(0.18–2.94)
Vo-tech or some college	1.86	(0.62–5.63)	0.99	(0.30–3.31)	3.19	(0.55–18.51)	0.40	(0.07–2.21)
College graduate or higher ®	1		1		1		1	
**Annual household income** (US$)								
≤25000	2.65	(0.98–7.15)	**10.96**	**(1.45– 82.61)**	1.33	(0.35–5.08)	5.82	(0.66–51.38)
25001–50000	1.92	(0.63–5.90)	3.20	(0.34–29.74)	0.59	(0.13–2.63)	2.34	(0.22–24.74)
>50000 ®	1		1		1		1	

AOR: adjusted odds ratio. ® Reference categories.

### Associations with quitting since the COVID-19 pandemic began

In bivariate analyses, only non-daily smoking was associated with quit attempts since the pandemic began (Table 4). No associations were found with quit attempts in multivariable analyses.

**Table 4:** Logistic regression showing associations between home smoking context, participant characteristics and any quit attempts since pre-COVID-19 among FQHC patients in rural Georgia

*Variables*	*Unadjusted (N=352)*		*Adjusted (N=223)*	
	*OR*	*95% CI*	*AOR*	*95 % CI*
**Number of smokers in home**	0.81	0.60–1.09	0.83	0.58–1.19
**Home smoking rules**				
Not allowed anywhere ®	1		1	
Allowed some places or some times	1.02	0.60–1.74	1.06	0.53–2.13
Allowed anywhere/no rules	0.83	0.51–1.35	0.69	0.34–1.39
**Stress level**				
Worse	1.28	0.82–1.98	1.34	0.76–2.39
Not worse ®	1		1	
**Smoking level**				
Daily smoking ®	1		1	
Non-daily smoking	1.93	1.02–3.63	1.95	0.86–4.42
**Sociodemographic covariates**				
**Sex**				
Male ®	1			
Female	1.04	0.67–1.61		
**Age** (years)	0.99	0.97–1.00		
**Marital status**				
Married or cohabiting ®	1		1	
Single	0.91	0.60–1.3	0.70	0.38–1.28
**Race/ethnicity**				
White ®	1		1	
Black/African American	1.13	0.73–1.75	0.93	0.51–1.68
**Education level**				
Some high school or lower	0.99	0.48–2.06	1.30	0.50–3.39
High school graduate or GED	1.61	0.80–3.25	1.38	0.56–3.42
Vo-tech or some college	0.93	0.43–1.99	1.07	0.41–2.80
College graduate or higher ®	1		1	
**Annual household income** (US$)				
≤25000	1.78	0.93–3.40	1.58	0.69–3.59
25001–50000	1.76	0.83–3.77	1.40	0.58–3.35
>50000 ®	1		1	

AOR: adjusted odds ratio. ® Reference categories.

## DISCUSSION

Slightly less than half of participants who smoke reported changing the amount they smoked due to the pandemic, with more reporting increased smoking than decreased. Prior research on the influence of the pandemic varies, with some studies showing a majority of smokers did not change their consumption levels, similar to our findings^5-8^, while others reporting higher proportions of either reduced^9-11^ or increased smoking^11-13^. Additionally, most participants did not change their cigarette purchasing behavior, and the majority did not change their levels of smoking inside the home, with <20% reporting either increases or decreases. Tattan-Birch et al.^21^ similarly found that the largest proportion of smokers neither increased nor decreased smoking inside their homes, although close to one-fourth either increased or decreased.

One of the primary objectives of our study was to assess whether household smoking context influenced smoking behaviors during the pandemic. As hypothesized, those who allowed smoking inside the home increased indoor smoking during the pandemic. However, we also observed that allowing smoking in the home was associated with reduced in-home smoking. It is possible that non-smoking family members encouraged smokers to smoke outside even without a formal rule to minimize exposure to secondhand smoke, given the increased togetherness in a closed environment during the pandemic. If true, future crises that require people to spend more time at home may provide an opportunity to promote smoke-free homes. Interestingly, the presence of young children was not associated with changes in cigarette smoking inside the home. It is difficult to compare our findings with others, given variations in research questions^6,21^. Driezen et al.^6^ examined whether more smokers permitted smoking in their homes in early 2020 relative to 2018, observing no difference in smoking allowed in the home, and no difference in the number of cigarettes smoked per day between the two time periods.

Consistent with our hypothesis, we found that the number of smokers in the home was associated with increased overall smoking levels during the pandemic. Still, it did not affect smoking inside the home or quit attempts. This aligns with research documenting that people who smoke tend to smoke more when around others who smoke^28^. However, combined with our findings on smoking in the home, this suggests that socially driven smoking may have occurred outside the home. The presence of multiple smokers in a household may create an environment where smoking is a shared activity, leading to increased consumption. However, this increased smoking may primarily occur outside the home, possibly due to household rules or a mutual understanding to minimize secondhand smoke exposure indoors. Additionally, during the pandemic, as suggested by our findings, individuals may have smoked more to manage increased stress, particularly in social settings with others who smoke, but without necessarily changing their indoor smoking habits due to existing restrictions or consideration for non-smoking household members. Lastly, the presence of other smokers in the household may have provided a network that reinforced smoking behavior, thereby not influencing quit attempts. This suggests that while social dynamics can increase smoking frequency, they may not consistently encourage smoking indoors or impact the motivation to quit.

As hypothesized, greater stress was associated with increased smoking (as shown in much of the existing research on smoking and COVID-19)^4,7,13,14,18^. Furthermore, it was associated with both increased and decreased smoking inside the home relative to no change. This dual association may be due to varying coping mechanisms among individuals: some may smoke more to manage stress, while others might reduce indoor smoking to protect household members from secondhand smoke during a stressful time. Additionally, we found no association between stress and quit attempts. The measure of stress we used in our models was general, in that it did not explore specific causes of stress (e.g. financial strain, sickness, or death of a loved one). Parsing out of actual causes of stress may have resulted in different findings and/or illuminated specific intervention targets for reducing stress in large-scale adversity (e.g. pandemics, natural disasters).

We also examined whether daily versus non-daily smoking affected smoking behavior during the pandemic. We found that non-daily smoking was associated with decreased smoking during the pandemic but not with quit attempts or levels of smoking in the home. Gravely et al.^5^ reported that smokers with lower dependence were more likely to try to quit or reduce smoking during the pandemic. Our results are consistent with this, as non-daily smokers were more likely to smoke less.

Several demographic characteristics that we examined were notable. Identifying as Black/African American was associated with both increased and decreased smoking inside the home. This variation might be due to differing household norms, levels of awareness about the risks of secondhand smoke, or varying stress-coping strategies. Being single was associated with lower odds of decreased smoking, which may be due to less pressure from household members to smoke less. Lower household income was associated with decreased smoking, likely due to financial pressures worsened by the pandemic, making it more challenging to afford cigarettes^4^.

### Limitations

Our results should be considered in light of several limitations. Most notably, our data were collected over a two-year enrollment period. Although COVID-19 remained a global pandemic throughout this data collection period, vaccines were rolled out, and most of the restrictions in place early in the pandemic, had been lifted by the time some of the study participants answered questions. To account for this, we compared responses on key variables by phase of the pandemic and detected no major patterns, so we pooled data across time for our analysis. Although this may make our findings harder to compare with other studies conducted early in the pandemic and often during the initial lockdown phase, the extended data collection period could be considered a strength, as it captured more enduring changes in behavior. Our data are self-reported and thus subject to social desirability and recall bias. Given the novelty of the situation, we also used newly created measures without psychometric testing. Additionally, participants were lower income primary care patients from largely rural regions of Georgia, and their experiences may not reflect those of persons who smoke in the general population.

## CONCLUSIONS

Our study reveals that the COVID-19 pandemic significantly influenced smoking behaviors among lower income, rural populations in Georgia, with nearly half of the persons who smoke altering their habits, predominantly increasing their smoking. Household smoking context and stress levels were key factors, as living with more smokers and allowing indoor smoking was linked to increased smoking. In contrast, worsened stress was associated with both increased and decreased smoking at home. Non-daily smokers were more likely to reduce their smoking, indicating lower dependence may facilitate behavior change under stress. Policymakers and tobacco control practitioners should be aware of the potential for increased smoking during future crises and be prepared to implement timely public health measures that can mitigate these effects. These include ensuring access to smoking cessation resources and support even during widespread disruption.

## Data Availability

The data supporting this research are available from the authors on reasonable request.
